# Paradoxical worsening of nontuberculous mycobacterial disease after the discontinuation of antitumor necrosis factor therapy: A case report

**DOI:** 10.1016/j.rmcr.2022.101599

**Published:** 2022-02-02

**Authors:** Daijiro Nabeya, Tomoo Kishaba

**Affiliations:** a Department of Infectious Diseases, Respiratory, and Digestive Medicine, Graduate School of Medicine, University of the Ryukyus, 207 Uehara, Nishihara, Okinawa, 903-0215, Japan; b Department of Respiratory Medicine, Okinawa Chubu Hospital, 281 Miyazato, Uruma, Okinawa, 904-2293, Japan

**Keywords:** Nontuberculous mycobacteria, *Mycobacterium avium* complex, Paradoxical response, Immune reconstitution inflammatory syndrome, Antitumor necrosis factor therapy, Tumor necrosis factor-α, TNF, tumor necrosis factor, IRIS, immune reconstitution inflammatory syndrome, NTM, nontuberculous mycobacteria, MAC, *Mycobacterium avium* complex, CT, computed tomography, HIV, human immunodeficiency virus

## Abstract

Antitumor necrosis factor–associated nontuberculous mycobacteria-immune reconstitution inflammatory syndrome (IRIS) has rarely been reported. An 84-year-old woman with a history of rheumatoid arthritis treated with etanercept was diagnosed with *Mycobacterium avium* complex (MAC) pulmonary disease six years before admission. Etanercept was discontinued two years ago because of MAC pulmonary disease progression and restarted nine months before admission because of worsening arthritis, again resulting in MAC pulmonary disease progression. Etanercept was discontinued again; however, the pulmonary disease progressed more rapidly. The condition was considered paradoxical worsening caused by IRIS due to etanercept discontinuation. The disease resolved quickly with chemotherapy for MAC.

## 1 Introduction

Tumor necrosis factor (TNF) inhibitors have recently played a pivotal role in treating autoimmune and inflammatory diseases. The use of anti-TNF therapy has increased the incidence of anti-TNF–associated tuberculosis [1]. It has further led to a rise in the cases of anti-TNF–associated tuberculosis-immune reconstitution inflammatory syndrome (IRIS) [2]. Moreover, the incidence of anti-TNF–associated nontuberculous mycobacteria (NTM) disease has increased [3]; however, anti-TNF–associated NTM-IRIS has rarely been reported.

The incidence of NTM diseases has recently been increasing worldwide [4]. Furthermore, there are no preventive recommendations for NTM diseases in patients treated with TNF inhibitors. Therefore, the clinical course of a patient with anti-TNF–associated NTM-IRIS would provide valuable information for clinicians. Herein, we report the case of a patient with anti-TNF–associated NTM-IRIS and present a review of the relevant literature.

## 2 Case presentation

The patient was an 84-year-old Japanese woman with a 42-year history of rheumatoid arthritis. She had started receiving methotrexate therapy 12 years before admission; etanercept, a TNF inhibitor, was added to the therapy ten years before admission. Six years before admission, she was diagnosed with *Mycobacterium avium* complex (MAC) pulmonary disease on the basis of a new lesion observed on her chest radiograph and sputum culture results, and it was observed because she remained asymptomatic and her MAC lesion was stable for some time. However, the pulmonary lesion expanded gradually, and etanercept was discontinued two years before admission due to onset of respiratory symptoms. Nine months before admission, etanercept was restarted owing to worsening arthritis; however, it was discontinued seven months before admission because of exacerbation of respiratory symptoms, such as cough and sputum. Even after etanercept discontinuation, home oxygen therapy for respiratory failure was started five months before admission. Chest computed tomography (CT) findings showed slight expansion of the MAC lesions in the lungs. Methotrexate was discontinued; however, the patient's clinical course continued to worsen. Finally, she visited the Chubu Hospital due to worsening hemoptysis and dyspnea.

**Fig. 1:**
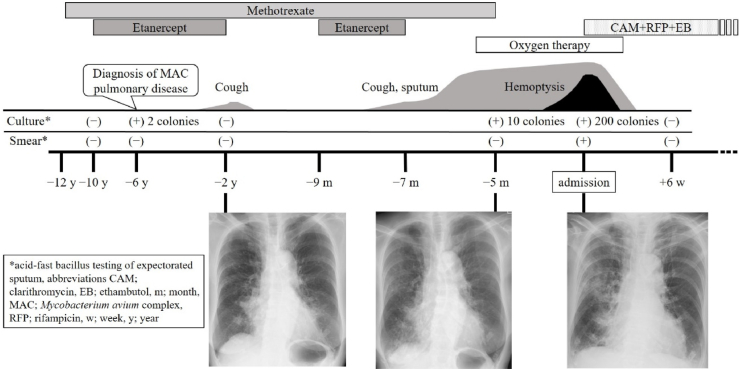
Clinical course and chest radiographs of the patient.

**Fig. 2:**
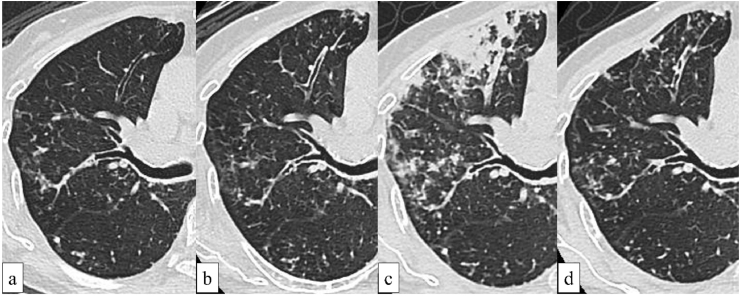
Chest computed tomography findings of the patient. **a.** Two years before admission, centrilobular nodules are observed in the right upper lobe. **b.** Five months before (two months after the discontinuation of the TNF inhibitor) admission, the centrilobular nodules have extended to the S3 region. **c.** On admission, the centrilobular nodules have increased in number and enlarged rapidly over five months. Furthermore, the lesions have aggregated and exhibit consolidation. **d.** Two months after starting chemotherapy, the consolidation has disappeared.

The clinical course and chest radiographs are shown in Fig. 1. Chest radiography on admission showed right upper lung field infiltration and a right lower lung field-dominant reticular shadow. Furthermore, chest CT showed worsening consolidation and centrilobular nodules in the right upper lobe (Fig. 2). Laboratory tests showed only a slight increase in C-reactive protein levels (1.42 mg/dL). The results of acid fast-bacilli smear and culture test of sputum revealed increased bacterial load. The MAC was identified to be *M. intracellulare/chimaera* using mass spectrometry. Chemotherapy for MAC (clarithromycin, rifampicin, and ethambutol) was initiated, resulting in an improvement in her respiratory symptoms within two weeks. Furthermore, her radiological findings promptly improved. Chemotherapy for MAC was discontinued six months later due to the appearance of refractory drug rashes. The MAC pulmonary disease has remained stable for over two years after chemotherapy.

Seven months before admission, the patient experienced clinical deterioration, even after etanercept discontinuation. Chest radiographs show lesion expansion in the right upper and lower lung fields. Additionally, the patient experienced chronic respiratory failure and required oxygen therapy. Finally, she was admitted because of worsening hemoptysis and respiratory failure. All clinical manifestations rapidly resolved after the initiation of chemotherapy.

## 3 Discussion

### 3.1 Clinical discussion

IRIS is a well-known complication observed in human immunodeficiency virus (HIV)-infected patients undergoing antiretroviral therapy and is defined in these patients as worsening of inflammatory symptoms during immune reconstitution due to antiretroviral therapy initiation [5]. Regarding IRIS in non-HIV-infected patients, there are several factors for IRIS onset, and anti-TNF discontinuation is one of the essential triggers [6]. TNF-α is a proinflammatory cytokine contributing to granulomatous inflammation, which is an essential immune response to AFB. Therefore, TNF inhibitor discontinuation can result in IRIS via rapid recovery of immune responses to AFB; a similar underlying mechanism is responsible for IRIS onset after antiretroviral therapy initiation in HIV-infected patients. In the present patient, clinical findings deteriorated right after the discontinuation of etanercept. CT morphology changed more rapidly than before the discontinuation of etanercept. No other pathogen was detected. Thus, the patient's condition was considered paradoxical worsening of MAC pulmonary disease caused by IRIS due to etanercept discontinuation.

TNF inhibitors are considered to increase the risk of NTM disease [3]; however, data from the United States Food and Drug Administration show that cases of anti-TNF–associated NTM diseases are reported less frequently than anti-TNF–associated tuberculosis [1]. The data shows that anti-TNF–associated NTM diseases occur less commonly compared to anti-TNF–associated tuberculosis. Moreover, anti-TNF–associated IRIS is a rare pathology; thus, anti-TNF–associated NTM-IRIS has rarely been reported. However, anti-TNF therapy is applied to many autoimmune diseases, and the incidence of NTM disease has been increasing globally [4]. Furthermore, there is no consensus statement on prophylaxis for NTM disease, unlike for the treatment of latent tuberculosis infection, at TNF therapy initiation. Clinicians must be careful about both NTM disease and NTM-IRIS in patients treated with TNF inhibitors.

### 3.2 Brief review of literature

The cases of anti-TNF–associated tuberculosis-IRIS have often been reported [2], whereas those of anti-TNF–associated NTM-IRIS are rare. In a previous case series of 13 patients with anti-TNF–associated NTM pulmonary disease, no patient was IRIS [7]. On searching the relevant literature, we found three case reports on anti-TNF–associated NTM-IRIS (Table 1). Patients whose cases were presented in these reports exhibited paradoxical progression of NTM disease after anti-TNF discontinuation, similar to the present patient. In the first case, a patient with Crohn's disease who was receiving infliximab developed disseminated MAC disease. Infliximab was discontinued and chemotherapy for MAC was initiated, resulting in an improvement in clinical findings. Although MAC pulmonary lesions expanded paradoxically three weeks later, the chemotherapy was completed without additional treatment [8]. In the second case, a patient with suspected spondyloarthritis who was receiving etanercept and methotrexate was diagnosed with *M. marinum* infection in the joints. Etanercept and methotrexate were discontinued, and chemotherapy was initiated; however, synovitis progressed within four months. Infliximab was eventually initiated to control inflammation. Subsequently, the treatment was successfully completed [9]. In the third case, a patient with relapsing polychondritis who was receiving adalimumab, tacrolimus, and prednisolone developed *M. intracellulare* pulmonary disease 16 months after the latest adalimumab administration. Tacrolimus was discontinued, and chemotherapy for MAC was initiated; however, the clinical condition worsened, and the patient developed disseminated MAC infection. Steroid dosage was increased, and treatment was subsequently completed [10]. Although the number of cases of anti-TNF–associated NTM-IRIS is insufficient, the clinical presentation of such patients might be mild compared to that of patients with anti-TNF–associated tuberculosis-IRIS [2]. Some of the patients from the reviewed case reports and the present patient had received cytotoxic drugs in addition to TNF inhibitors. Increased bacterial load due to the use of multiple immunosuppressants might have caused IRIS onset. Additionally, the clinical condition of the patient in the third case report progressed to IRIS right after the discontinuation of the cytotoxic drug, whereas anti-TNF therapy had been discontinued over a year before IRIS onset [10]. The third case findings suggest that cytotoxic drug discontinuation also triggers IRIS onset.

**Table 1:** Summary of case reports on anti-TNF–associated NTM-IRIS.

Case no.	Pathogen	Underlying disease	Immunosuppressant treatment	NTM disease-affected organ	IRIS-affected organ	Additional treatment	Outcome
1 [7]	*M. avium* complex	Crohn's disease	Infliximab	Lungs	Lungs	None	Resolved
2 [8]	*M. marinum*	None	Etanercept + methotrexate	Joints	Joints	Infliximab	Resolved
3 [9]	*M. intracellulare*	Relapsing polychondritis	Adalimumab + prednisolone + tacrolimus	Lungs	Lymph nodes, subcutaneous tissue	Increased corticosteroid dosage	Resolved
Present patient	*M. intracellulare/chimaera*	Rheumatoid arthritis	Etanercept + methotrexate	Lungs	Lungs	None	Resolved

TNF, tumor necrosis factor; NTM, non-tuberculous mycobacteria; IRIS, immune reconstitution inflammatory syndrome.

Patients with anti-TNF–associated tuberculosis-IRIS sometimes treated successfully with the administration of corticosteroids and/or the re-administration of TNF inhibitors as anti-inflammatory agents [2]. The reviewed case reports show that patients with refractory anti-TNF–associated NTM-IRIS were also successfully treated with corticosteroid administration or TNF inhibitor re-administration [9,10]. Therefore, these treatment options may be used for patients with refractory anti-TNF–associated NTM-IRIS.

## 4 Conclusion

• Paradoxical worsening of NTM pulmonary disease in the presented patient was considered IRIS due to etanercept discontinuation
• Although anti-TNF–associated NTM-IRIS is rare, clinicians should consider the possibility of IRIS when NTM diseases worsen in patients receiving anti-TNF therapy

## Funding

This report did not receive any specific grant from funding agencies in the public, commercial, or not-for-profit sectors.

## Ethics

There are no ethical issues regarding this manuscript. The medical record of the patient was retrospectively reviewed and identifying information removed.

## Author contributions

Conceptualization - D.Nabeya.

Data curation - D.Nabeya.

Formal analysis - D.Nabeya.

Funding acquisition - None.

Investigation - D.Nabeya.

Methodology - D.Nabeya.

Project administration – T.Kishaba.

Resources – D.Nabeya, T.Kishaba.

Software – None.

Supervision – T.Kishaba.

Validation – D.Nabeya, T.Kishaba.

Visualization - D.Nabeya.

Roles/Writing - original draft - D.Nabeya.

Writing - review & editing – D.Nabeya, T.Kishaba.

## Declaration of competing interest

None.
